# A portable optical-fibre-based surface plasmon resonance biosensor for the detection of therapeutic antibodies in human serum

**DOI:** 10.1038/s41598-020-68050-x

**Published:** 2020-07-07

**Authors:** Luigi Zeni, Chiara Perri, Nunzio Cennamo, Francesco Arcadio, Girolamo D’Agostino, Mario Salmona, Marten Beeg, Marco Gobbi

**Affiliations:** 1 Department of Engineering, University of Campania Luigi Vanvitelli, Via Roma 29, 81031 Aversa, Italy; 2 Copernico SRL, Via Monte Hermada, 75, 33100 Udine, Italy; 3 Department of Biochemistry and Molecular Pharmacology, Istituto Di Ricerche Farmacologiche Mario Negri IRCCS, Via Mario Negri 2, 20157 Milan, Italy

**Keywords:** Optics and photonics, Imaging and sensing, Optical spectroscopy

## Abstract

Different lines of evidence indicate that monitoring the blood levels of therapeutic antibodies, characterized by high inter-individual variability, can help to optimize clinical decision making, improving patient outcomes and reducing costs with these expensive treatments. A surface plasmon resonance (SPR)-based immunoassay has recently been shown to allow highly reliable and robust monitoring of serum concentrations of infliximab, with significant advantages over classical ELISA. The next level of advancement would be the availability of compact and transportable SPR devices suitable for easy, fast and cheap point-of-care analysis. Here we report the data obtained with recently developed, cost-effective, optical-fibre-based SPR sensors (SPR-POF), which allow the construction of a compact miniaturized system for remote sensing. We carried out an extensive characterization of infliximab binding to an anti-infliximab antibody immobilized on the SPR-POF sensor surface. The present proof-of-principle studies demonstrate the feasibility of the proposed SPR-POF platform for the specific detection of infliximab, in both buffer and human serum, and pave the way for further technological improvements.

## Introduction

Surface plasmon resonance (SPR), which is based on the interaction of light and free electrons in the semi-transparent noble metal layer placed on a dielectric substrate, is one of the most sensitive and commonly used techniques for monitoring interactions between two unlabelled molecules^1^. Thus, the binding of an analyte, present in solution, to its receptor-ligand immobilized on the metal surface results in the local change of the refractive index (RI) of the surrounding medium, which in turn affects the electromagnetic wave propagating along the metal–dielectric interface in a highly sensitive manner.

Up to now, several SPR sensor configurations have been developed. Classical prism-based sensors coupled with microfluidic systems^2–4^ have been classically used to measure the binding constants underlying the analyte-ligand interaction, due to the possibility of following the association and dissociation rate constants, and thus the equilibrium dissociation constant, in real time.

SPR-based assays may also be very useful for rapid quantification of analyte concentrations^5,6^, as confirmed recently during the characterization of an SPR-based method for the measurement of the serum concentrations of infliximab (IFX), a therapeutic anti-TNFα antibody widely used to treat chronic inflammatory diseases^7^. The availability of a rapid and reliable method for the monitoring of the blood levels of therapeutic antibodies, characterized by high inter-individual variability, can help to optimize clinical decision making and rational use of these expensive treatments^8–14^. The SPR-based assays offer significant advantages over classic ELISA, in particular avoiding the long incubation/separation/washing/detection steps, reducing complexity and variability^7,15^. However, the implementation of SPR-based monitoring at the point-of-care, just before the antibody infusion, requires the availability of SPR instruments that are simpler and cheaper than the conventional ones.

Optical-fibre-based SPR sensors have been developed first using silica optical fibres and then polymer optical fibres. These sensors don’t need expensive optical equipment and allow a compact miniaturized system and remote sensing capability^16^. Several applications in environmental, industrial, biological and medical fields have been demonstrated by coating optical-fibre SPR sensors with antibodies, aptamers or synthetic bio-mimetic polymers^17–20^. In the framework of rapid, simple to make and to use, and low-cost systems based on optical fibre sensors, polymer optical fibres (POF) are increasingly preferred due to advantageous properties such as simple handling, excellent flexibility, robust and compact construction, low cost, high numerical aperture, large diameter and the ability to withstand smaller bending radii than glass^21,22^. The main drawback of the polymer optical fibres is the inability to use them in environments where high temperature (higher than 80 °C) is reached, due to the damage that can be caused to the fibre itself. However, these high temperatures are not reached in usual conditions where biosensors are employed and, should they be reached, the bio-interfaces (proteins, antibodies, etc.) or bio-mimetic interfaces (for example molecularly imprinted polymers) will fail as well. The failure of the bio-interfaces means that glass fibre-based sensors will not work in high-temperature settings as well.

In this work, we analysed the potential of an SPR-POF biosensor, based on a transmission mode set-up, for the specific detection of IFX in human serum. The exploited platform, extensively described in Cennamo et al.^23^, lends itself very well to be coupled with different molecular recognition elements, including antibodies^24^, aptamers^25,26^ and molecularly imprinted polymers^27,28^. These previous applications have shown the real capabilities of this simple and low-cost approach in bio-sensing and chemical sensing, demonstrating its reliability.

The SPR-POF platform is created from a D-shaped POF. The POF’s exposed core was covered through the deposition of a photoresist layer and a thin gold layer. The POF-sensor is then coated with a specific anti-IFX antibody for IFX detection. The obtained experimental results and their comparison with a conventional SPR instrument^7^ demonstrated that this POF biosensor can be used in therapeutic drug monitoring (TDM) applications.

Lu et al.^29,30^ presented a similar approach using glass optical fibres and a reflection-mode SPR set-up, where the SPR signal was amplified, exploiting gold nanoparticles combined with the gold film and the self-assembling monolayer of the bioreceptor, to improve the performance. A performance comparison is reported in the discussion section.

## Materials and methods

### Chemicals and reagents

IFX (Remicade) was purchased from MSD Italia S.r.l. (Rome, Italy). The anti-IFX antibody HCA-216 was purchased from Bio-Rad Laboratories (Segrate, Italy). Control IgG was purchased from Merck Life Science S.r.l. (Rom, Italy), and 10 × Dulbecco-PBS was obtained from Euroclone S.p.A. (Pero, Italy). Tween 20 and α-lipoic acid were obtained from Sigma-Aldrich (Milan, Italy). Water was provided in-house by a Milli-Q system (Millipore, Bedford, MA, USA).

Anonymized human serum samples from blood donors were obtained from Mario Negri Biobank ‘Saturne’ Certified ISO 9001:2015, a non-profit service unit aimed at the collection and conservation of human blood samples for scientific research purposes in accordance with the Helsinki Declaration and the Italian legislation (Ministerial Decree of 15 July 1997 and subsequent updates).

### SPR assay using a conventional, prism-based instrument

The ProteOn XPR36 protein interaction array system (Bio-Rad Laboratories, Hercules, CA, USA)^3^ was used for these studies. Lipoic acid was coupled to the bare gold surface of a BGD sensor chip (BioRad) using the same protocol used for the SPR-POF instrument (see below). Briefly, the chip was taken out of the housing and immersed for 18 h at 25 °C in a freshly prepared solution of 40 mM α-lipoic acid dissolved in 10% ethanol. Then, the surface was washed three times with Milli-Q water, dried under a gentle nitrogen stream, put into the chip housing and mounted onto the instrument. Anti-IFX antibody and IgG (as reference) were then immobilized on the lipoic acid self-assembled monolayer using amine-coupling chemistry. The surface was activated with 0.04 mM sulfo-NHS/0.3 mM EDC mixture, according to the manufacturer’s directions to form N-hydroxysuccinimide esters. The ligands were diluted in PB buffer (pH 7.4), at a concentration of 30 µg/mL. These solutions were then flowed for 5 min at a rate of 30 µL/min over the activated chip surface. The remaining activated groups were blocked with 1 mM ethanolamine, pH 8.0.

After rotation of the fluidic system, IFX solutions (83–1,333 ng/mL) were injected in parallel channels, so that they flowed over the anti-IFX antibody and IgG, and, in parallel, on the lipoic acid surface for reference. The running buffer was 10 mM phosphate buffer containing 150 mM NaCl and 0.005% Tween 20 (PBST pH 7.4). The IFX solutions flowed over immobilized ligands for 3 min at a rate of 30 µL/min. Dissociation was measured in the following 10 min. All these assays were done at 25 °C. The sensorgrams [time course of the SPR signal in resonance units (RU)] were normalized to a baseline value of 0. The signals observed on the surfaces coated with the anti-IFX antibody were corrected by subtracting the nonspecific response observed in the surfaces with IgG immobilized.

### Plasmonic sensor system: SPR optical fibre sensor platform and experimental set-up

The optical sensor developed by modifying a plastic optical fibre (POF) with a core of poly-methylmethacrylate (PMMA) of 980 µm and a cladding of fluorinated polymer of 10 µm, as already described in^23^, is briefly recalled here. First, a D-shaped POF region was obtained by removing the cladding and part of the core, along half the circumference, by a very simple polishing process, using two different kinds of polishing papers^23^. Second, a photoresist layer was spun (6,000 rpm for 60 s) on the exposed POF core with the use of a spin coater machine. More specifically, Microposit S1813 photoresist, which has a refractive index higher than the one of the POF cores, was used to improve both the performances and the adherence of the gold film^23^.

The spin coating technique allowed the deposition of a uniform layer of about 1.5 µm thickness of the photoresist (buffer layer). Finally, a thin gold film was sputtered by a Bal-Tec SCD 500 machine. The sputtering process was repeated three times by applying a current of 60 mA, at 0.05 mbar of pressure, for 35 s to obtain a 60 nm thick layer (20 nm of gold for step). The above-described sensor had a sensing region (the D-shaped POF area) of 10 mm in length.

The equipment needed to obtain the intrinsic optical fibre sensor configuration was simple and low-cost, including a halogen lamp in input and a spectrometer in output. The halogen lamp HL-2000-LL (manufactured by Ocean Optics, Dunedin, FL, USA) used as the white light source had an emission range from 360 to 1,700 nm. The spectrometer (FLAME-S-VIS-NIR-ES, manufactured by Ocean Optics, Dunedin, FL, USA) had a detection range from 350 nm to 1,023 nm. The above-described sensor was connected to the light source and the spectrometer by two SMA connectors.

The transmission spectra, along with data values, were displayed online on the computer screen and saved with software provided by Ocean Optics, setting the integration time at 1,000 µs and the averaging of the scans at 150. The SPR transmission spectra, achieved with air as surrounding medium and normalized to the reference spectrum, were obtained using the Matlab software (MathWorks, Natick, MA, USA). The Hill fittings of the experimental values were obtained through OriginPro software (Origin Lab. Corp., Northampton, MA, USA).

### Immobilization process on the SPR-POF platform surface

According to the immobilization procedure reported in Cennamo et al.^31^, the POF surface was sequentially cleaned with Milli-Q water (3 times for 5 min) and 10% ethanol solution in Milli-Q water (3 times for 5 min). Then the gold surface was treated in three different steps: (1) production of a surface exposing carboxylic acids, using α-lipoic acid, (2) activation of this surface with sulfo-N-hydroxysuccinimide/1-ethyl-3-(3-dimethylaminopropyl)-carbodiimide (sulfo-NHS/EDC), and (3) covalent immobilization of the anti-IFX antibody by amine-coupling chemistry.

In the first step, the gold chip was immersed for 18 h at 25 °C in a freshly prepared solution of 40 mM α-lipoic acid dissolved in 10% ethanol. Then, the surface was washed three times with milli-Q water, and the chip was incubated for 20 min at room temperature with a mixture of sulfo-NHS/EDC (200 mM and 50 mM, respectively), in potassium phosphate buffer (50 mM, pH 7.4). After this step, the surface was incubated for 2 h at room temperature with 0.1 mg/mL of the anti-IFX antibody (100 μL) dissolved in sodium phosphate buffer (10 mM, pH 7.4). The remaining activated carboxyl groups were blocked by exposing the surface with 1 M ethanolamine, pH 8.0, for 30 min at room temperature.

At the end of this treatment, the chips were washed with sodium phosphate buffer (10 mM) containing 150 mM NaCl and 0.005% Tween 20 (PBST pH 7.4) and finally dried under a nitrogen flow.

The effectiveness of the immobilization procedure could be observed by measuring the difference between the resonance wavelength before and after the immobilization process with a buffer solution without the analyte. In this case, the measured shift (due to refractive index variation) depends only on the gold surface chemically modified through the formation of a self-assembling monolayer (SAM). When the SAM is present, the refractive index of the dielectric layer in contact with the gold surface increases, and so the resonance wavelength shifts to the right^25,26^.

### Binding experiments exploiting the SPR-POF platform

All experiments were performed using the same protocol: about 70 µL of IFX solution (in buffer or diluted human serum) were dropped over the sensing region of the SPR-POF sensor and incubated at room temperature for ten minutes to let the interaction between the immobilized receptor (i.e. the anti-IFX antibody) and analyte (i.e. IFX) occur. At the end of this incubation, a washing step with PBST was performed, and the spectrum was recorded. By adopting this protocol, only the shift of the resonance wavelength determined by the specific binding analyte-receptor was measured, eliminating shifts due to bulk changes or non-specific interactions. The binding between the receptor on the SPR-POF platform and the analyte, both in buffer and in real matrix, was evaluated in the range from 0 to 50,000 ng/mL. Saturation curves were fitted according to the “One site – Specific binding” model provided by Prism (GraphPad Prism 8.4), to obtain the equilibrium dissociation constant (Kd) and the maximum binding (Bmax) with their 95% confidence intervals (CI). Extra sum of-squares F-test included in the GraphPad Prism software was used to check the statistical significance of the differences between Kd values, *p* < 0.05 was considered significant.

## Results

### Preliminary studies with a conventional, prism-based SPR instrument

Preliminary studies were carried out using the Proteon XPR36 SPR instrument (Biorad), which permits following what happens on the sensor surface during the different experimental steps in real time. We used a bare-gold BGD chip pre-functionalized (out of the instrument) with lipoic acid to expose carboxylic acid moieties on the chip surface.

**Figure 1:**
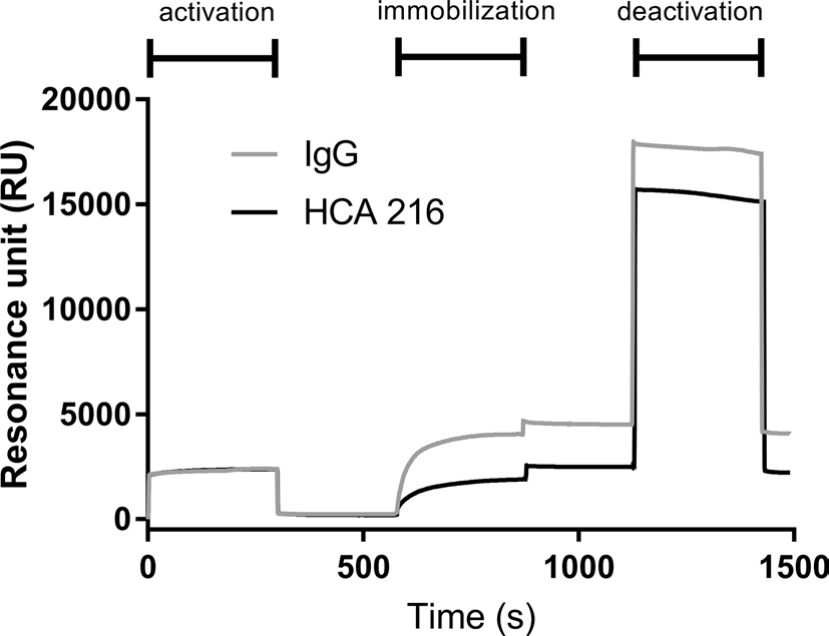
Effects of the three steps of the immobilization procedure as evaluated in real time in the ProteonXPR36 SPR instrument. For these studies, we used a bare gold chip pre-coated (out of the instrument) with lipoic acid. The figure shows the changes in the SPR signals (in resonance units) during the sequential 5 min flow with EDC/NHS (activation), followed by IgG or the anti-IFX antibody (immobilization) and ethanolamine (deactivation). The SPR signal measured at the end of the whole procedure indicates the immobilization level.

The chip was inserted in the instrument, and the following solutions were flowed in sequence: (1) EDC/NHS (activation step), (2) anti-IFX antibody or IgG (immobilization step) and (3) ethanolamine (deactivation step). The SPR signals measured in real time during these steps are shown in Fig. 1, which also shows that the immobilization levels of the anti-IFX antibody and IgG at the end of the procedure were 2,214 RU and 4,077 RU, respectively, thus indicating the effectiveness of this immobilization procedure.

**Figure 2:**
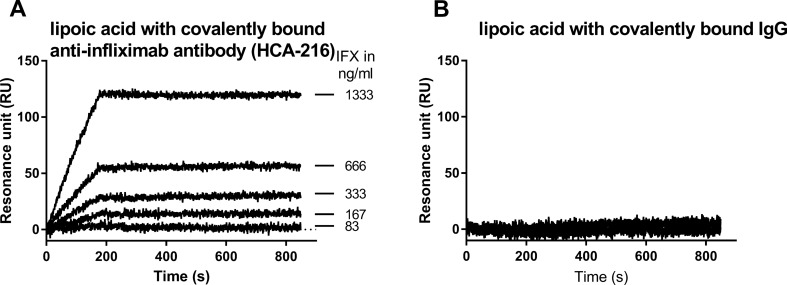
SPR sensorgrams obtained with the ProteOn XPR36 instrument by flowing IFX at different concentrations in PBST pH 7.4 over immobilized anti-IFX (**A**) or the control surface (**B**, IgG only). These sensorgrams were corrected by the signal obtained on the reference with lipoic acid only.

Concentration-dependent binding of IFX was observed when flowing the antibody over the immobilized anti-IFX antibody (Fig. 2A). In contrast, no binding was observed on the control surface (Fig. 2B, with IgG only).

### Studies with an SPR-POF platform

**Figure 3:**
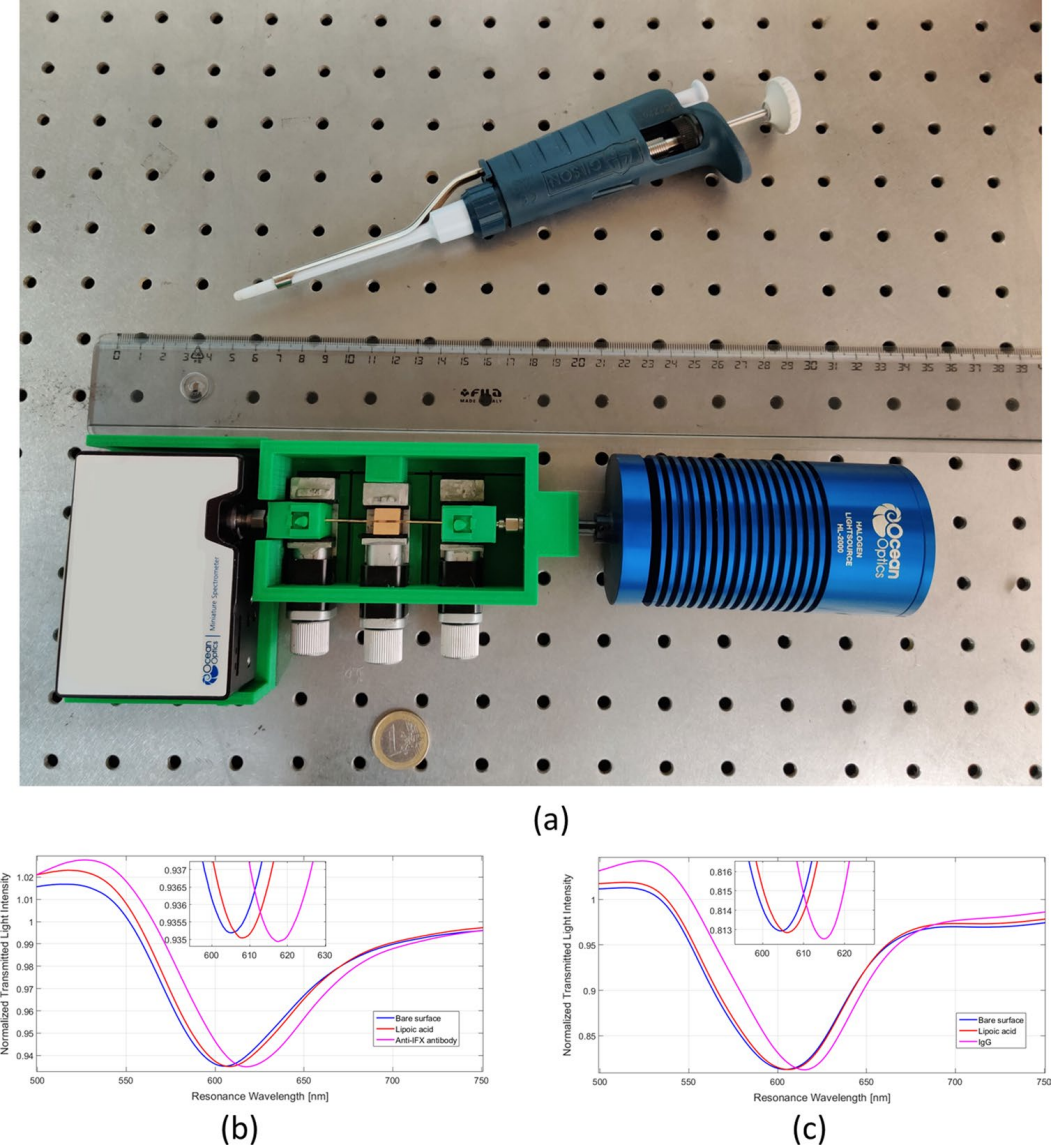
(**a**) Picture of the SPR-POF sensor system; (**b**, **c**) resonance spectra of gold surface functionalization process, obtained by SPR-POF platform in buffer solution (PBST), before and after the functionalization process with lipoic acid on the gold surface followed by immobilization with the anti-IFX antibody (**b**) or IgG (**c**).

Once the effectiveness of the experimental procedures was validated with the conventional SPR instrument, the following studies were carried out with the SPR-POF platform shown in Fig. 3a. This POF biosensor system is a simple and transportable device suitable for easy, fast and cheap point-of-care analysis. The sensor system size is small compared to a bench-top conventional instrument: maximum width 10 cm; maximum length 35 cm; and maximum height 4 cm. Moreover, the sizes of the spectrometer and the light source could be further reduced, even if their price increases. At the same time, the limit of detection of the biosensor could be improved by replacing the POF platform with tapered POFs or/and exploiting gold nanoparticles, as described below.

In this section, we first report on the monitoring of the functionalization step and then on the SPR responses observed after binding of the analyte to its specific receptor (both in buffer and serum).

#### Functionalization of the gold surface

Figure 3b, c shows the spectra obtained in buffer before and after the procedure for the immobilization of (1) lipoic acid on the gold surface of the SPR-POF platform and (2) anti-IFX antibody or IgG on the lipoic acid surface.

These spectra indicate a certain shift of the resonance wavelength toward higher values at the end of the procedure for lipoic acid immobilization, and a further, more marked shift after immobilization of anti-IFX antibody or IgG.

A typical shift, associated with a SAM of bio-receptor on the SPR-POF platform of this kind, ranges from 5 to 20 nm, as previously found^25,26^. In this work, the shift was about 10 nm.

#### Infliximab detection in PBST buffer

**Figure 4:**
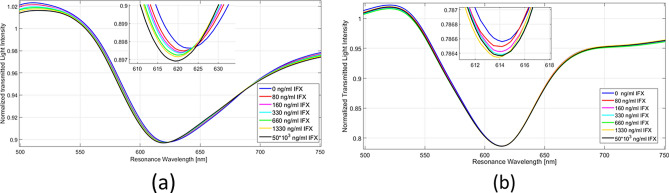
SPR spectra obtained by exposing the SPR-POF functionalized with the anti-IFX antibody (**a**) or IgG (**b**) to different concentrations of infliximab (IFX) in buffer (PBST). These spectra were normalized to the reference spectrum.

Figure 4 shows the normalized transmission spectra of the SPR-POF with anti-IFX antibody (a) or IgG as reference (b), after the incubation with increasing concentrations of IFX in the buffer (PBST). These data indicate that IFX induces a concentration-dependent shift of the resonance wavelength to lower values only in the presence of immobilized anti-IFX antibody, but not with immobilized IgG. These data indicate that the binding of IFX causes a decrease of the refractive index of the immobilized anti-IFX antibody. The decrease of medium’s refractive index due to the presence of IFX has also been tested by a refractometer (SPR-POF sensor with a bare gold surface), as shown in Figure S1.

**Figure 5:**
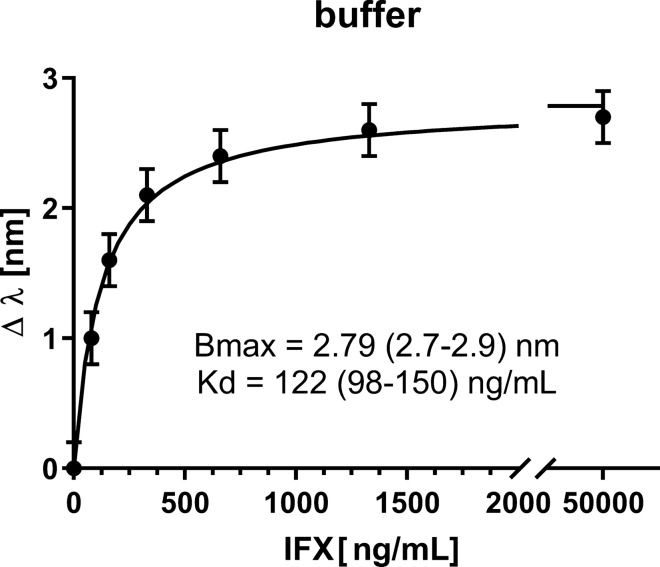
Concentration-dependent effects of IFX (in PBST) on the SPR wavelength shifts (Δλ) using an SPR-POF surface-functionalized with the anti-IFX antibody. The experimental data were fitted by non-linear regression using the ‘one-site’ binding model, i.e. the simplest model describing the equilibrium binding of a ligand (IFX) to a receptor (anti-IFX antibody) as a function of increasing ligand concentration. The best-fit values of the model parameters (Bmax and Kd), are indicated with their 95% confidence interval values (in parenthesis). The error bars correspond to the upper bound of the experimentally measured standard deviation.

The IFX-dependent shifts (Δλ) observed on the immobilized anti-IFX antibody can be well fitted using the equation of a simple binding isotherm or saturation binding curve, which assumes the presence of a simple 1:1 interaction (Fig. 5). More specifically, Fig. 5 shows the resonance wavelength variation (Δλ) versus the IFX concentration (ng/mL), the error bars and the fitting to the experimental data. In particular, each experimental point is the average of five subsequent measurements and the error bars represent the upper bound of the standard deviation. These data strongly support the point that the shifts are due to the binding of IFX to immobilized anti-IFX antibodies. Because a clear saturation was observed with the IFX concentrations used, a Kd value could be reliably estimated consistent with a very high affinity (122 ± 11 ng/mL, corresponding to 0.81 ± 0.07 nM).

The limit of detection (LOD), estimated by extrapolating from the curve the IFX concentration corresponding to three times the standard deviation of the blank (λ_0_, 0.15 nm), was 23.5 ng/mL, i.e. similar, or even lower, than the one obtained with the conventional SPR instrument (100 ng/mL).

#### Infliximab detection in real matrix

**Figure 6:**
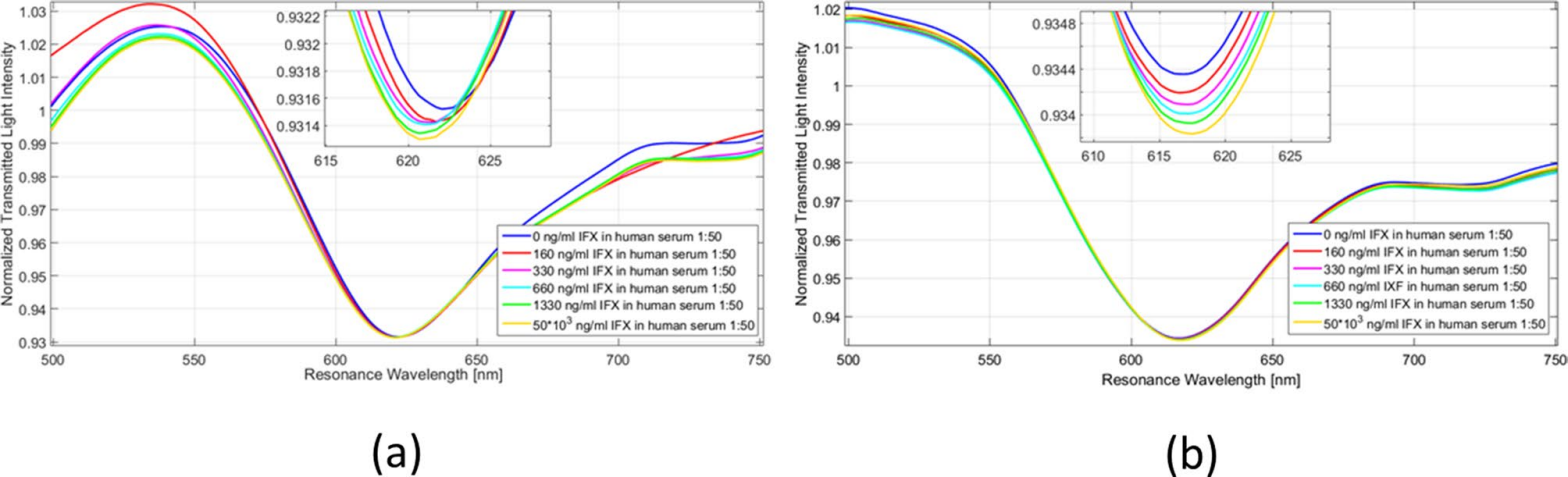
SPR spectra obtained exposing the SPR-POF functionalized with the anti-IFX antibody (**a**) or IgG (**b**) to different concentrations of infliximab (IFX) in human serum diluted 50-fold. These spectra were normalized to the reference spectrum.

**Figure 7:**
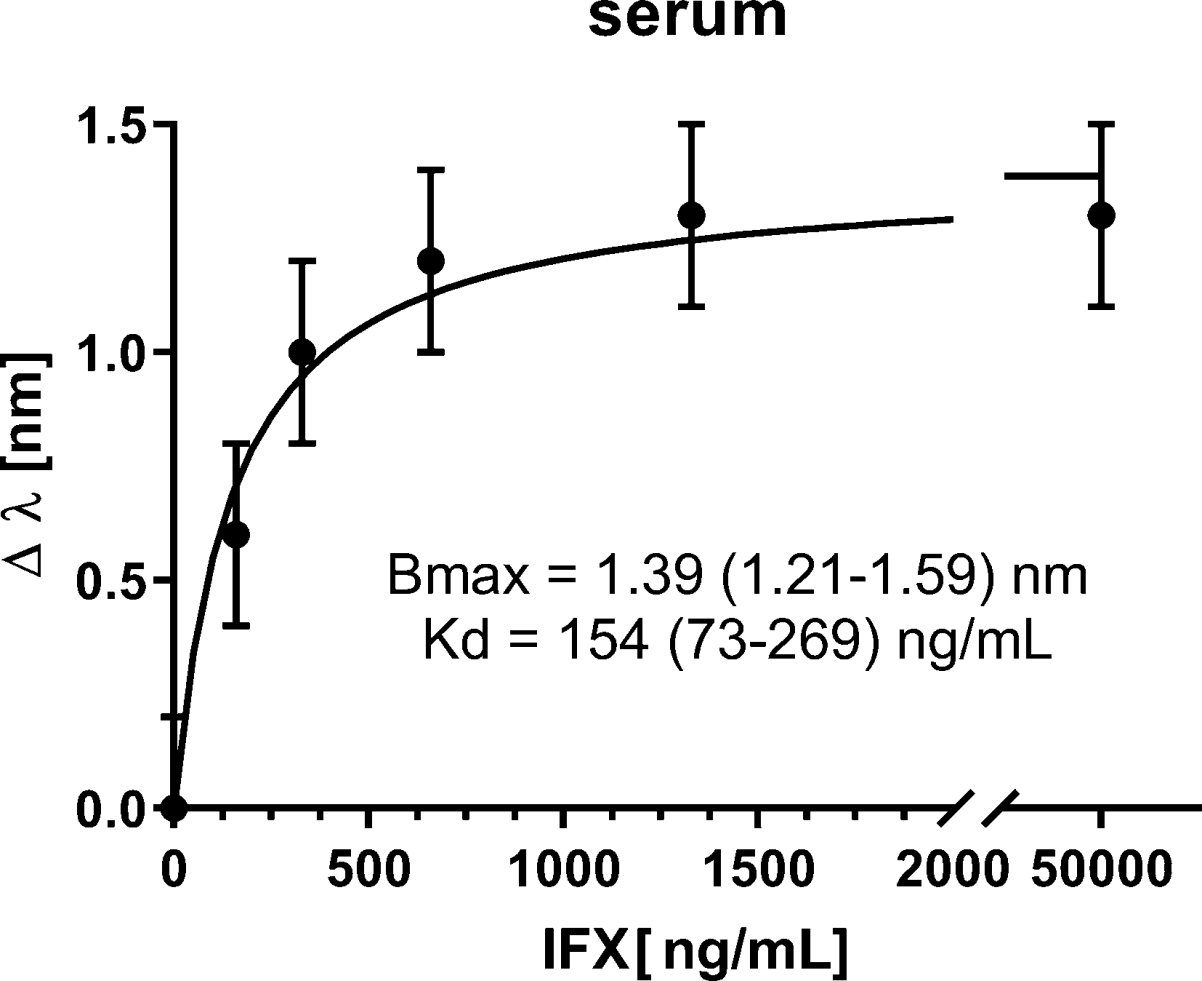
Concentration-dependent effects of IFX (in human serum diluted 50-fold) on the SPR wavelength shifts (Δλ) using an SPR-POF surface functionalized with anti-IFX antibody. The experimental data were fitted by non-linear regression using the ‘one-site’ binding model, i.e. the simplest model describing the equilibrium binding of a ligand (IFX) to a receptor (anti-IFX antibody) as a function of increasing ligand concentration. The best-fit values of the model parameters (Bmax and Kd) are indicated with their 95% confidence interval values (in parenthesis). The error bars correspond to the upper bound of the experimentally measured standard deviation.

Following studies were then carried out spiking IFX in the real matrix, i.e. human serum. Preliminary analyses highlighted non-specific bulk effects induced by serum alone, which could be satisfactorily minimized by 50-fold dilution of this matrix. Using the SPR-POF functionalized with the anti-IFX antibody, we confirmed the IFX-induced shifts of the resonance wavelengths to lower values (Fig. 6a). This effect was concentration-dependent (Figs. 6a, 7) and specific as indicated by the lack of IFX effect on SPR-POF functionalized with generic IgGs (Fig. 6b).

In this case, too, the IFX concentration-dependent shifts (Δλ) observed on the immobilized anti-IFX antibody could be well fitted using the equation of a simple binding isotherm (Fig. 7). Although the maximal effect was about 50% lower than that seen in buffer (compare with Fig. 3), probably because of a matrix-dependent quenching, the Kd in serum (154 ± 37 ng/mL, corresponding to 1.03 ± 0.25 nM) was similar to that in buffer. The two values were not significantly different as determined by the extra sum-of-squares F-test included in the GraphPad Prism software.

The LOD, estimated by extrapolating from the curve the IFX concentration corresponding to three times the standard deviation of the blank (λ_0_, 0.15 nm), resulted in 73.7 ng/mL, i.e. similar to the one obtained with the conventional SPR instrument (80 ng/mL)^7^.

## Discussion and conclusion

Therapeutic drug monitoring (TDM) is increasingly proposed to guide therapy with biologics, to optimize dosage regimens or change the therapeutic approach, thus improving patient outcomes and reducing costs. Recent data from our laboratory suggested that SPR-based immunoassay may allow highly reliable and robust monitoring of serum concentrations of infliximab^7^ and other therapeutic antibodies (data not published), with significant advantages over classical ELISA. These studies were carried out with a conventional SPR instrument, suitable for centralized analysis only. The possibility to have a compact and transportable SPR-based device, allowing reliable, easy, fast and cheap analysis at the point-of-care, i.e. just before the clinical decision, would significantly boost the applicability and the usefulness of TDM.

The present studies were undertaken to verify the potential of such a device, relying on SPR and polymer optical fibres, to measure clinically relevant concentrations of IFX in human serum, as a proof-of-principle for TDM application. As with the conventional instrument, used in parallel for comparison, the IFX interaction with anti-IFX antibodies induced the SPR phenomenon on the sensor surface. Control surfaces were prepared by immobilization of IgGs. The results demonstrated that IFX is detectable by the SPR-POF device in a specific and concentration-dependent manner, both in buffer and human serum, with LOD of 23.5 and 73.5 ng/mL respectively. These results are similar to the LOD obtained with the conventional SPR instrument, ProteOn XPR36.

Recently, silica fibre optic SPR (FO-SPR)-based immunoassays were proposed to detect IFX and adalimumab (another anti-TNFa therapeutic antibody) in human serum^29,30,32^. As in our SPR-POF setting, anti-drug antibodies were immobilized on the FO probe as capture agents. However, unlike in our setting, the direct exposure of the FO-SPR probe to the therapeutic antibodies did not result in measurable SPR signals, thus requiring a signal amplification with a sandwich design (with gold nanoparticles functionalized with another set of IFX specific antibodies). Although this amplification step allowed very low LOD (2.2 ng/mL in serum, perhaps unnecessarily low considering the clinically relevant therapeutic range), it represents a further incubation passage that is not needed with our device^29,30^. In fact, the direct measurement of drug binding to its ligand, instead of using secondary antibodies, can be advantageous, especially when anti-drug antibodies are measured^7^. Furthermore, plastic optical fibres are much cheaper than silica fibres, enabling better optimization of the costs in developing point-of-care devices in the future. Although the LOD obtained by this POF biosensor (73.7 ng/mL of diluted serum) is still not enough to measure the lowest clinically relevant concentrations of IFX (500 ng/mL serum, i.e. 10 ng/mL in 50-fold diluted serum), these proof-of-concept studies provide the basis for further optimisation of the set-up. It is worth noting that the LOD of our device is comparable with that of standard, bulky and expensive, laboratory SPR instruments, when the same surface layer (lipoic acid) and ligand (anti-IFX) are used, thus suggesting the possibility to reach the sensitivity required for a therapeutic drug monitoring^7^. With this aim, we plan to increase the surface density of anti-IFX, or to use TNFα as the immobilized ligand since our data with the conventional SPR indicate that the combination of IFX/TNFα results in a better sensitivity than the combination IFX/anti-IFX^7^. Moreover, Cennamo et al. demonstrated that it is possible to achieve higher sensitivity by using a tapered POF^33^, accepting a more complex manufacturing procedure and a higher cost. Alternatively, to further improve the performances of the proposed SPR-POF sensor platform, the D-shaped tapered POF could be combined with nano-particles, as was already demonstrated with a synthetic receptor (molecularly imprinted polymer) directly incorporating gold nano-stars, so allowing the excitation of localized surface plasmon resonance (LSPR) and increasing the sensitivity accordingly^34^. Of course, also this last approach requires a more complex manufacturing procedure which increases the costs.

The future developments of this POF biosensor will also require significant advances in the miniaturization and full integration of the POF probe with inexpensive optoelectronics devices to obtain a low-cost and small size biosensor system, with the possibility of connection to the Internet (exploiting the capability of the so-called ‘Internet of Things’).

## Supplementary information

Supplementary information
