# Supplementary material for: A portable optical-fibre-based surface plasmon resonance biosensor for the detection of therapeutic antibodies in human serum
Source: Sci Rep. 2020 Jul 7;10:11154. doi: 10.1038/s41598-020-68050-x (PMC7341820; doi:10.1038/s41598-020-68050-x)
Supplement: Supplementary file 1 — Supplementary information [file 41598_2020_68050_MOESM1_ESM.docx]

**A PORTABLE OPTICAL-FIBER BASED**

**SURFACE PLASMON RESONANCE BIOSENSOR FOR THE**

**DETECTION OF THERAPEUTIC ANTIBODIES IN HUMAN SERUM**

Luigi Zeni^§^°, Chiara Perri^§^, Nunzio Cennamo^§^, Francesco Arcadio^§^, Girolamo D’Agostino^§§^, Mario Salmona^§§§^, Marten Beeg^§§§^, Marco Gobbi^§§§^°

§ Department of Engineering

University of Campania Luigi Vanvitelli

Via Roma 29

81031 Aversa - ITALY

§§ Copernico SRL

Via Monte Hermada, 75

33100 Udine - ITALY

§§§ Department of Biochemistry and Molecular Pharmacology

Istituto di Ricerche Farmacologiche Mario Negri IRCCS

Via Mario Negri 2

20157 Milano - ITALY

° corresponding authors (e-mail: [luigi.zeni@unicampania.it](mailto:luigi.zeni@unicampania.it), marco.gobbi@marionegri.it)

**Supplementary Information**

The shift of the resonance wavelength to lower values, due to the presence of infliximab, was even confirmed by dropping a concentrated solution of the analyte in MilliQ-water on the bare gold surface of the SPR-POF platform, i.e., just using the platform as a refractometer. This means that the presence of infliximab in water reduces the refractive index, as it occurs in the bio-receptor. The resonance curves showing the above effect are reported in Figure S1: the resonance wavelength is shifted to lower values, indicating a decrease of the refractive index, when the infliximab is present in the water.


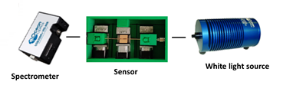

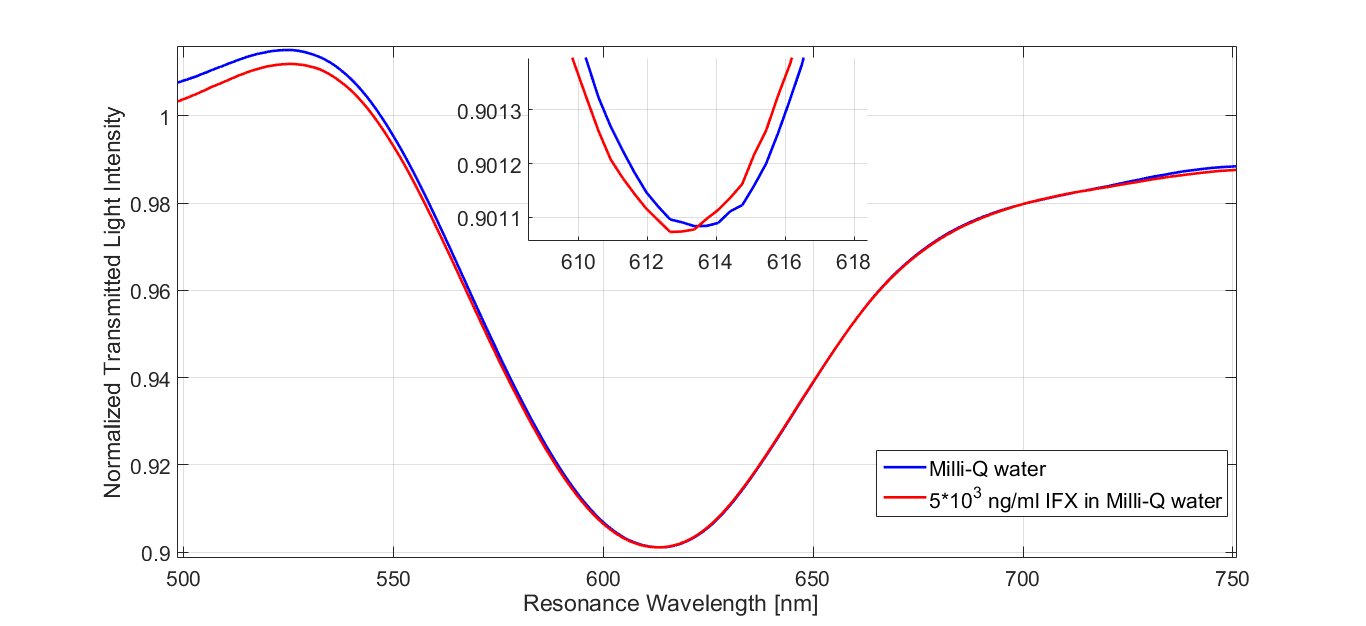


**Figure S1.** SPR spectra obtained in Milli-Q water and in a Milli-Q water solution of IFX on the bare gold surface of the SPR-POF sensor.
